# Capturing sequence variation among flowering-time regulatory gene homologs in the allopolyploid crop species *Brassica napus*

**DOI:** 10.3389/fpls.2014.00404

**Published:** 2014-08-25

**Authors:** Sarah Schiessl, Birgit Samans, Bruno Hüttel, Richard Reinhard, Rod J. Snowdon

**Affiliations:** ^1^Department of Plant Breeding, IFZ Research Centre for Biosystems, Land Use and Nutrition, Justus Liebig University, GiessenGiessen, Germany; ^2^Max Planck Genome Centre Cologne, Max Planck Institute for Breeding ResearchCologne, Germany

**Keywords:** copy number variation, CNV, presence-absence variation, PAV, rapeseed, sequence capture

## Abstract

Flowering, the transition from the vegetative to the generative phase, is a decisive time point in the lifecycle of a plant. Flowering is controlled by a complex network of transcription factors, photoreceptors, enzymes and miRNAs. In recent years, several studies gave rise to the hypothesis that this network is also strongly involved in the regulation of other important lifecycle processes ranging from germination and seed development through to fundamental developmental and yield-related traits. In the allopolyploid crop species *Brassica napus*, (genome AACC), homoeologous copies of flowering time regulatory genes are implicated in major phenological variation within the species, however the extent and control of intraspecific and intergenomic variation among flowering-time regulators is still unclear. To investigate differences among *B. napus* morphotypes in relation to flowering-time gene variation, we performed targeted deep sequencing of 29 regulatory flowering-time genes in four genetically and phenologically diverse *B. napus* accessions. The genotype panel included a winter-type oilseed rape, a winter fodder rape, a spring-type oilseed rape (all *B. napus* ssp. *napus*) and a swede (*B. napus* ssp. *napobrassica*), which show extreme differences in winter-hardiness, vernalization requirement and flowering behavior. A broad range of genetic variation was detected in the targeted genes for the different morphotypes, including non-synonymous SNPs, copy number variation and presence-absence variation. The results suggest that this broad variation in vernalization, clock and signaling genes could be a key driver of morphological differentiation for flowering-related traits in this recent allopolyploid crop species.

## Introduction

As a recent allopolyploid species, *Brassica napus* L. (genome AACC, 2*n* = 38) is also a very interesting model to investigate polyploidization and adaptation during crop evolution. Although oilseed rape/canola (*B. napus* ssp. *napus*) is today the second-most important oilseed crop worldwide, it is thought that the species originated only during the last few thousand years, after spontaneous interspecific hybridization events between Asian *Brassica rapa* (genome AA, 2*n* = 20) and Mediterrranean *Brassica oleracea* (genome CC, 2*n* = 18) (Snowdon et al., 2006).

No wild forms of *B. napus* are known, and intensive selection and breeding following its anthropogenically-influenced polyploidization has led to cultivation of very different phenological types. This has caused the diversification of distinct gene pools adapted to highly different eco-geographic zones of Europe, Asia/Australia and North America. Very early-flowering morphotypes, without vernalization requirement, are today widely grown in Canada (as canola) and northern Europe (as spring oilseed rape), where harsh winters prohibit autumn-sown crops. Later-flowering “semi-winter” oilseed forms, requiring only mild vernalization, are prevalent in China and Australia, while autumn-sown oilseed rape is today the most important oilseed crop in temperate regions of Europe (Friedt and Snowdon, 2010). The subspecies *napus* also includes leafy forms that sometimes need strong vernalization before flowering and are grown in parts of Europe and eastern Asia as fodder rape or kales. A second subspecies, *B. napus* ssp. *napobrassica*, comprises swede forms with an enlarged hypocotyl that is harvested as a vegetable or used as a grazing fodder. Swedes generally have a strong vernalization requirement but tend to lack the strong winter-hardiness of winter oilseed rape (Friedt and Snowdon, 2010).

*Brassica napus* is the most closely related major field crop species to the model crucifer *Arabidopsis thaliana*. This enables considerable insight into major biochemical and developmental pathways using information from the model species. For example, important *Brassica* orthologs of *A. thaliana* genes responsible for vernalization and floral transition are highly conserved between the model and the crop (Lagercrantz et al., 1996; Osborn et al., 1997; Wang et al., 2009; Zou et al., 2012). In Arabidopsis, the optimization of flowering in respect to environment is achieved by a tightly regulated gene network determining the transition from the vegetative to the reproductive phase (Jaeger et al., 2006; Jung and Muller, 2009; Srikanth and Schmid, 2011; Andrés and Coupland, 2012). There is increasing evidence that this network not only regulates flowering time *per se*, but also plays a role throughout the whole plant life cycle (Deng et al., 2011). The pleiotropic or direct influence of flowering time regulators on multiple agronomic traits, like the number and size of seeds, seedling vigor, biomass gain and resistance to biotic or abiotic stress (Quijada et al., 2006; Chen et al., 2007; Ni et al., 2008; Chianga et al., 2009; Basunanda et al., 2010; Li et al., 2010), not only makes them a major driver of crop evolution and adaptation, but also subjects them to strong selection for useful diversity during crop breeding.

To meet the needs of their respective climate zone, plants developed several sensor systems to assess the correct time to flower. Of particular importance in this regard is an ability to sense temperature, day length, light quality and stress signals (Jaeger et al., 2006; Jung and Muller, 2009; Srikanth and Schmid, 2011; Wigge, 2013). In temperate climates zones where winter limits growth completely, the most important mechanism of plant flower regulation is vernalization, the induction of flowering after a period of prolonged cold (Preston and Sandve, 2013). The second condition for plants to flower after winter is day length (Song et al., 2013), whereas light quality and other forms of stresses can only modulate the flowering response. Moreover, the transition to flowering can also be influenced by endogenous factors like gibberellins and autonomous pathways like the circadian clock (Pak et al., 2009; de Montaigu et al., 2010). Understanding the role and interplay of these factors could assist in improving yield and adaption in *B. napus*.

**Figure 1:**
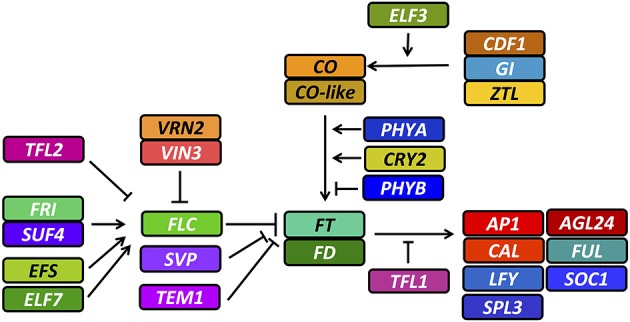
**Overview of relevant flowering time genes and their interactions in *A. thaliana***. Full gene names and descriptions of their interactions are given in the text. Arrows indicate positive regulation, whereas blunt ends indicate repression.

Knowledge of flowering in *Brassica* species is largely based on *A. thaliana*. The most important Arabidopsis genes involved in flowering time have already been shown to have orthologs in *Brassica* crops (Wang et al., 2009; Zou et al., 2012), whereby comparisons of *A. thaliana* and *B. rapa* suggest that this congruence might be true for the whole flowering-time gene network (http://brassicadb.org/brad/flowerGene.php#). In *A. thaliana* the network features two major thresholds controlling the main flowering signal, *FLOWERING LOCUS T (FT)*. The first threshold, the vernalization pathway, acts via removal of a factor repressing *FT* expression upon perception of the stimulus, while the second threshold, the photoperiod pathway, acts via *FT* activation. Repression of *FT* in the vernalization pathway is achieved by several factors, the most important being *FLOWERING LOCUS C (FLC)*, assisted by other factors like *SHORT VEGETATIVE PHASE (SVP)* and *TEMPRANILLO 1 (TEM1). FLC* is constitutively expressed before vernalization by activation of *FRIGIDA (FRI*), which acts in complex with other factors like *SUPPRESSOR OF FRIGIDA 4 (SUF4*) as a transcriptional activator for *FLC*. Expression of *FLC* is also enhanced by other factors like *EARLY FLOWERING 7 (ELF7)* and *EARLY FLOWERING IN SHORT DAYS (EFS)*. The signal for FLC silencing is transmitted via upregulation of *VERNALIZATION INSENSITIVE 3 (VIN3)* in response to prolonged cold. VIN3 binds to a complex named PCR2, a major component of this complex being *VERNALIZATION 2 (VRN2)*. The PCR2 complex is associated with the *FLC* gene segment and silences *FLC* transcription by heterochromatic changes upon binding of *VIN3*. During this process, *TERMINAL FLOWER 2 (TFL2)* also binds to the *FLC* gene and may be responsible for conserving the vernalized state. *FLC* is then effectively silenced and not responsive to further activation by the *FRI* complex, making *FT* accessible for activation by the photoperiod pathway. *FT* is activated by the transcription factor *CONSTANS (CO)*, which is only stably expressed at the end of a long day. This expression pattern is controlled by the circadian clock, transmitting its signal via *GIGANTEA (GI)* in complex with *ZEITLUPE (ZTL)*, and *CYCLING DOF FACTOR 1 (CDF1*). This transmission is also modulated by ambient temperature via *EARLY FLOWERING 3 (ELF3)*. Protein stability of *CO* is further controlled by photoreceptors. *PHYTOCHROME A (PHYA)* and *CRYPTOCHROME 2 (CRY2)* stabilize CO protein, whereas *PHYTOCHROME B (PHYB)* destabilizes it. As soon as vernalization and photoperiod pathway allow for FT expression, FT is translocated to the shoot apex, triggering the vegetative-to-generative transition in a complex with *FLOWERING LOCUS D (FD)*, via direct or indirect activation of several meristem identity genes like *APETALA 1 (AP1)* and *CAULIFLOWER (CAL)*. These are further modulated by an interwoven network of transcription factors including the miRNA-regulated *SQUAMOSA PROMOTER BINDING PROTEIN-LIKE 3 (SPL3), AGAMOUS-LIKE 24 (AGL24), LEAFY (LFY), FRUITFUL (FUL)* and *SUPPRESSOR OF CONSTANS 1 (SOC1)*. The function of FT is antagonized by *TERMINAL FLOWER 1 (TFL1)*, which contributes to the fine regulation of flowering time in response to ambient temperature, independently from vernalization (reviewed in depth in Jaeger et al., 2006; Jung and Muller, 2009; Pak et al., 2009; de Montaigu et al., 2010; Srikanth and Schmid, 2011; Wigge, 2013) (summarized in Figure 1).

Despite the common ancestry and functionality of most genes, a major difference between the genetic control particularly of complex traits in *B. napus* and *A. thaliana* is the influence of polyploidy. The progenitor genomes making up the *B. napus* genome are still basically intact (Parkin et al., 1995; Axelsson et al., 2000; Bancroft et al., 2011). This means that every gene may have homologous alleles present in the A genome as well as in the C genome, which are hard to differentiate from alleles within each subgenome (Bancroft et al., 2011). Furthermore, each gene may have multiple paralogs within each subgenome as a consequence of whole-genome triplication and gene duplication in the diploid species (Town, 2006; Wang et al., 2011b). Gene expression studies revealed an average of 4.4 functional gene copies present in *B. napus* (Parkin et al., 2010). Furthermore, during allopolyploidization the two *B. napus* subgenomes frequently exchange gene material in a process called homoeologous recombination (Gaeta and Pires, 2010; Udall et al., 2006). This dynamic genome formation process has been shown to result in gene copy-number variation (CNV) and presence/absence variation (PAV), affecting traits with relevance for adaptation, selection and breeding (Harper et al., 2012). The extent of such variants within high-impact regulatory networks like the circadian clock, vernalization and floral transition pathways has yet to be investigated in the context of adaptive traits in *B. napus*.

Analysis of the *B. rapa* genome sequence has also revealed an expansion of transposons (Wang et al., 2011b). Transposons play a major role in creating genetic variation, the most important prerequisite of adaptation (reviewed in Lisch, 2012). Recently it was shown that a Tourist-like MITE insertion in the promoter region of a *FLOWERING LOCUS C* (*FLC*) homolog in the *B. napus* A-genome is associated with vernalization requirement in European winter rapeseed morphotypes (Hou et al., 2012). Transposal activity is highly accelerated in case of genomic shock caused by interspecific hybridization and chromosomal breakage (Lisch, 2012), both of huge relevance in *Brassica* species. As much as 8–15% of the *B. napus* genome is comprised of repetitive sequences, indicating a potentially high degree of transposal activity (Samans, unpublished data). As the C genome is larger than the A genome (Johnston, 2005), transposons might be expected to be more prominent in C-genome gene homologs.

Generally, polyploids are considered to be more stable and adapt easier to new environments (Chen, 2010). One reason is the number of gene copies, which can be a regulating factor. More copies offer the possibility of (1) simultaneous transcription, therefore accelerating or strengthening regulation responses, (2) separate regulation in order to reach a more elaborate fine-tuning, and (3) gene back-ups to reduce loss-of-function risks. In plants, copy number variation has been observed to be wide-spread (Żmieńko et al., 2013). Due to the high degree of genome and gene duplication and genome rearrangements during polyploid formation, a relatively high degree of copy number variation is expected in *B. napus* (Edwards et al., 2013). All the same, classical cloning and mapping strategies suffer from this complexity. Due to the high specificity of cloning, it is often not possible to evaluate the number of copies present in a genome without exact knowledge from a high-quality reference genome.

As a proof of principle, this study aimed to detect genetic variation in all homologous and paralogous copies of 29 selected flowering time genes in *B. napus*, based on sequences derived from the diploid progenitors *B. rapa* and *B. oleracea*. Four genotypes representing the broad phenological variation for vernalization requirement, flowering transition and day-length dependent flowering in *B. napus* were sequenced with an RNA-based sequence capture approach. The objectives were (1) to establish an effective RNA bait library for sequencing of flowering time regulatory genes in the allopolyploid *B. napus*, (2) to investigate gene losses and gains amongst flowering-related genes in different *B. napus* ecotypes, and (3) to determine the extent of genetic variation among flowering time and vernalization pathway genes in *B. napus*.

## Materials and methods

### Plant material

A large panel of genetically diverse *B. napus* inbred lines was previously tested for winter survival, date of flowering and duration of flowering under short and long day conditions. The plant material used to select the different morphotypes was the ERANET-ASSYST consortium diversity set, a panel of over 500 genetically diverse *B. napus* accessions described in (Bus et al., 2011; Körber et al., 2012). The panel was grown either in full or in part at a number of different locations in Germany from 2009 until 2013, in southwest China from 2011 to 2013 and in central Chile from 2012 until 2013. In Germany, where winters generally have prolonged periods with temperatures well below freezing, accessions requiring vernalization and known to have moderate or good winter survival (“winter-type” rapeseed) were grown in autumn-sown trials (sowing in late August or early September, with harvest the following July). A large panel of swede genotypes, which require vernalization before flowering but generally have considerably lower winter survival, were also grown in the autumn-sown trials. Spring-type accessions with poor winter survival and no vernalization requirement were grown in Germany in spring-sown trials (sowing in March or April, harvest generally around September). In Temuco, central Chile, where the winter is mild but has a sufficient cold period for vernalization of *B. napus*, the winter-type and spring-type accessions were grown together in a spring-sown trial to differentiate photoperiod sensitive flowering after short, mild vernalization. The winter-type and spring-type accessions were also grown in Chongqing, southwestern China, where the winter is mild and day-length variation is considerably less extreme than in northern Europe.

Based on the results of these field studies, an initial screening panel comprising four *B. napus* ideotypes with considerable phenological variation in terms of vernalization requirement, winter survival, flowering time and photoperiod sensitivity, was selected for the sequence capture experiment. The four selected genotypes were: (1) the winter-hardy, vernalization requiring but late-flowering winter oilseed rape “25629-3,” (2) the winter-hardy, vernalization requiring but early-flowering fodder rape “Silona,” (3) the winter-sensitive, spring-type canola “Campino,” which requires no vernalization and exhibits day-length dependent flowering (all *B. napus* ssp. *napus*), and (4) the swede “Magres Pajberg” (*B. napus* ssp. *napobrassica*), which has a low winter survival but requires vernalization and flowers very late.

Homozygous inbred lines of the four accessions were generated by self-pollination to at least the S5 generation over many years. Leaf material for genomic DNA extraction was harvested from each accession in spring 2012 from field trials performed in Giessen, Germany. Mixed leaf samples were taken from at least 5 different plants, immediately shock-frozen in liquid nitrogen and kept at −20°C until extraction.

### DNA isolation

Leaf material was ground with a mortar and pestle under liquid nitrogen. DNA was extracted using a common CTAB protocol modified from Doyle and Doyle (1990). Fifteen milli liter of hot (65°C) extraction buffer (1.4 M NaCl, 50 mM Cetyltrimethylammoniumbromid (CTAB), 50 mM Na_2_S_2_O_5_, 0.1 M Tris/HCl pH 8.0, 20 mM EDTA, 30 mM mercaptoethanol) were added to 2 g of frozen ground leaf material, vortexed and incubated for 30 min at 65°C in a water bath. 15 ml of chloroform-isoamylalcohol (24:1, v/v) were added and mixed for 5 min at room temperature by inverting the tube. The mixture was centrifuged (Beckmann Coulter Allegra X-30R, [S/N 13D 1125], 3400 rpm, 4°C, 10 min) and the supernatant was transferred to a second tube. 12 ml of chloroform-isoamylalcohol (24:1, v/v) were added and again mixed for 5 min. The sample was centrifuged as before and the supernatant was transferred to a third tube. For precipitation of the amino acids 1 ml each of 3 M NaOAc and 10 M NH_4_OAc were added together with cold (4°C) isopropanol in a volume of 2/3 of the supernatant. DNA was then separated by centrifugation (Beckmann Coulter Allegra X-30R, (S/N 13D 1125), 3000 rpm, 4°C, 10 min) and the pellet was washed in 500 μl washing ethanol (70% (v/v) ethanol, 10 mM NH_4_OAc). The washed pellet was dried and diluted in TE buffer (10 mM Tris/HCl pH 8.0, 1 mM EDTA). 10 μl RNase A (1 mg/ml) per 100 μl TE were added and incubated for 16 h at room temperature. 3 M NaOAc and 10 M NH_4_OAc were added to a volume of 10 μl each per 100 μl TE, followed by 80 μl isopropanol per 100 μl TE. The resulting pellet was separated again by centrifugation (sigma 2K15 (12148), 8000 rpm, 4°C, 10 min) and washed in 500 μl washing ethanol. The washed pellet was dried and diluted in the same amount of TE. DNA concentration was determined using a Qubit fluorometer and the Qubit dsDNA BR assay kit (Life Technologies, Darmstadt, Germany) according to the manufacturer's protocol. DNA quantity and purity was further checked on 0.5% agarose gel (3V/cm, 0.5xTBE, 120 min).

### Selection of target genes

A set of 29 flowering time genes was selected based on literature from *A. thaliana* and the *Brassicaceae*. The genes were selected to cover the entire genetic network controlling flowering time, including circadian clock regulators (*CDF1, ELF3, GI*, and *ZTL*), the input pathways for vernalization (*ELF7, EFS, FLC, FRI, SVP, SUF4, TFL2, VRN2, VIN3*), photoperiod sensitivity (*CO, CRY2, PHYA, PHYB*) and gibberellin (GA3ox1), along with downstream signal transducers (*AGL24, AP1, CAL, FD, FT, FUL, LFY, SPL3, SOC1, TEM1, TFL1*).

### Retrieval of gene sequences for bait development

Full-length *A. thaliana* genomic sequences from all of the target genes were retrieved from NCBI. Because no reference genome for *B. napus* was available at the time of the bait construction, orthologous copies of the genes in the *Brassica* A genome were identified in the reference sequence of *B. rapa* using “synteny search” and “non-synteny search” at the database *BRAD* (http://brassicadb.org/brad/ accessed in June 2012). For homologs in the C genome, both *A. thaliana* and *B. rapa* sequences were blasted against the *B. oleracea* sequence database *bolbase* (http://www.ocri-genomics.org/bolbase/ accessed in June 2012). The BLAST settings were: database: B.oleracea.v.1.0.DNA, blastn (Default settings). Every hit with an *E*-value of e^−50^ or lower was taken into account. Full genomic sequences for the identified *B. oleracea* genes were kindly provided by Professor Shengyi Liu, Oil Crops Research Institute, Chinese Academy of Agricultural Sciences, Wuhan, China.

Full genomic sequences for 6 *B. napus* copies of *FT* were provided by Carlos Molina, Christian Albrechts University, Kiel, Germany. One copy (*Bna.FT.A02*) included the promoter sequence. Full genomic sequences for two copies of *Bna.CO* were retrieved from NCBI (GenBank accession numbers AF016011.1 and AF016010.1).

### Bait development

120mer oligonucleotide sequences were developed using the Agilent Genomic Workbench program eArrayXD (Agilent Inc., Santa Clara, CA, USA; https://earray.chem.agilent.com/earray/helppages/index.htm#earrayxd_and_the_earray_web_site.htm). For *B. rapa*, the reference sequence file from *BRAD* was loaded as custom genome. Alongside the full *B. rapa* reference genome sequence (v 1.1), each of the retrieved gene sequences was loaded into eArrayXD as a pseudo-chromosome to generate a custom reference for bait generation from the target genes.

Bait groups were created in eArrayXD using the “Bait Tiling” tool. The parameters were set as follows: Sequencing Technology: “Illumina,” Sequencing Protocol: “Paired-End long Read (75 bp+),” “Use Optimized Parameters (Bait length 120, Tiling Frequency 1x),” Avoid Overlap: “20,” “User defined genome,” “Avoid Standard Repeat Masked Regions.” The strand was selected manually depending on the location of the respective gene. Baits for genes on the minus-strand were developed in sense, while baits on the plus-strand were developed in antisense.

In total, 64 bait groups were created for *B. rapa* copies of the target genes, 68 bait groups for *B. oleracea* copies and 8 bait groups for *B. napus* copies.

### Sequence capture and sequencing

Custom bait production was carried out by Agilent Technologies using the output oligonucleotide sequences from eArrayXD. Sequence capture was performed using the SureSelectXT 1 kb-499 kb Custom Kit (Agilent Inc., Santa Clara, CA, USA) according to the manufacturer's instructions. The resulting TruSeq DNA library (Illumina Inc., San Diego, CA, USA) was sequenced on an Illumina HiSeq 2500 sequencer at the Max Planck Institute for Breeding Research (Cologne, Germany) in 100 bp single read mode.

### Data analysis

Quality control of the raw sequencing data was performed using FASTQC. Reads were mapped onto a pre-publication draft (version 4) of the *B. napus* “Darmor-Bzh” reference genome sequence assembly, which was kindly made available prior to public release by INRA, France, Unité de Recherche en Génomique Végétale (Boulos Chaloub, INRA-URGV, Èvry, France, unpublished data). Mapping was performed using the SOAPaligner algorithm (http://soap.genomics.org.cn/soapaligner.html) with Default settings and the option r=0 to achieve uniquely aligned reads. Removal of duplicates, sorting and indexing was carried out with *samtools* version 0.1.19 (http://samtools.sourceforge.net/). Alignments were visualized using the IGV browser version 2.3.12 (http://www.broadinstitute.org/igv/). Enriched regions and coverage differences were calculated using the *bedtools* software genomeCoverageBed (http://bedtools.readthedocs.org/en/latest/) with the option –bg. Calling of single nucleotide polymorphisms (SNPs) was performed with the algorithm mpileup in the *samtools* toolkit. Calling of insertions/deletions (InDels) was performed with SOAPInDel and results of InDel mapping were compared using Bowtie2 (2.1.0, http://bowtie-bio.sourceforge.net/bowtie2/index.shtml). Predicted sequences of the target genes in the *B. napus* Darmor-Bzh genome were annotated with BLAST2GO and used for comparisons with enriched positions. The target was defined using BLAST positions of respectively annotated genes and the bait pool (*E*-value cut-off e^−100^) on the mapping reference, und used for fraction calculation.

Read coverage for each captured region was normalized as follows: coveragenorm = (number of reads of equally covered region*total length of genome)/(number of aligned reads*read length). Copy number variation (CNV) in a given target region was assumed if the ratio of normalized coverage(genotype)/normalized coverage(all genotypes) was smaller than 0.5 or higher than 1.5, respectively. Presence/absence variation (PAV) was assumed if the ratio was smaller than 0.05.

Gene coding sequences and translated peptide sequences were determined using GENSCAN (http://genes.mit.edu/GENSCAN.html), with settings for “Arabidopsis.” The translated sequences were aligned to available protein sequences for *B. napus*, *B. rapa*, *B. oleracea*, and *A. thaliana* using the software CLC SequenceViewer (CLC Genomics, Aarhus, Denmark). Analysis of promoter regions was also done with CLC SequenceViewer. Sequences were aligned with gap open cost = 10, gap extension cost = 1 and settings of “very accurate,” first in subgroups aligning to the closest public sequence and then as a total to allow alignment in different regions. From this alignment, a neighbor joining tree was constructed with bootstrapping, using Default settings.

## Results

### Sequence capture

Using the *aligner* algorithm of SOAP2, 83–88% of all sequence reads could be aligned successfully for the four accessions. Table S1 lists alignment results for the four genotypes. As expected, the reads from the winter oilseed 25629-3, which is the most closely related of the four accessions to the reference genotype Darmor-Bzh, showed the highest alignment rates. The lowest alignment rates were seen in the swede Magres Pajberg, which represents the divergent subspecies *B. napus* ssp. *napobrassica*. The alignment success was independent of the total number of reads.

The number of aligned reads per library varied from around 3 million (Campino) to over 13 million (Magres Pajberg), allowing us to test the effect of different levels of target coverage on the detection of additional homoeologous loci, CNV and PAV. The normalized mean coverage of the total targeted sequence regions ranged from 879 times (879x) to 985x, with a target size of 614 kbp. Between 72 and 76% of the target was sequenced with a minimum coverage of 10 reads (equivalent to 0.2–0.5% of the genome). Between 19 and 22% of the intended target sequence was not captured, indicating a capture sensitivity (the fraction of target covered) of 78–81%. The ratio of absolute mean coverage in the target to total mean coverage suggests an enrichment factor of more than 760x. The two genotypes with over 10 million reads showed only a slightly higher fraction of covered target sequence than those sequenced with 3–5 mio reads. The specificity (fraction of reads covering the target) was also found to vary only slightly, from 50 to 52% (Table 1).

**Table 1:** **Coverage and genomic fractions of aligned reads in respect to target**.

**Sequence coverage**	**25629-3**	**Silona**	**Campino**	**Magres Pajberg**
Mean genome-wide coverage	0.47	1.35	0.38	1.48
Mean target coverage	362.19	1042.21	306.11	1150.26
Enrichment factor	767.20	773.41	802.27	779.80
Normalized mean target coverage	918.54	879.46	985.55	904.12
Fraction of target covered (%)	81.40	81.37	78.45	79.12
Reads covering target (%)	51.13	51.93	51.72	50.45
Genome fraction covered by >10 reads (%)	0.28	0.42	0.24	0.45
Target fraction covered by > 10 reads (%)	75.90	76.61	71.90	73.30

**Figure 2:**
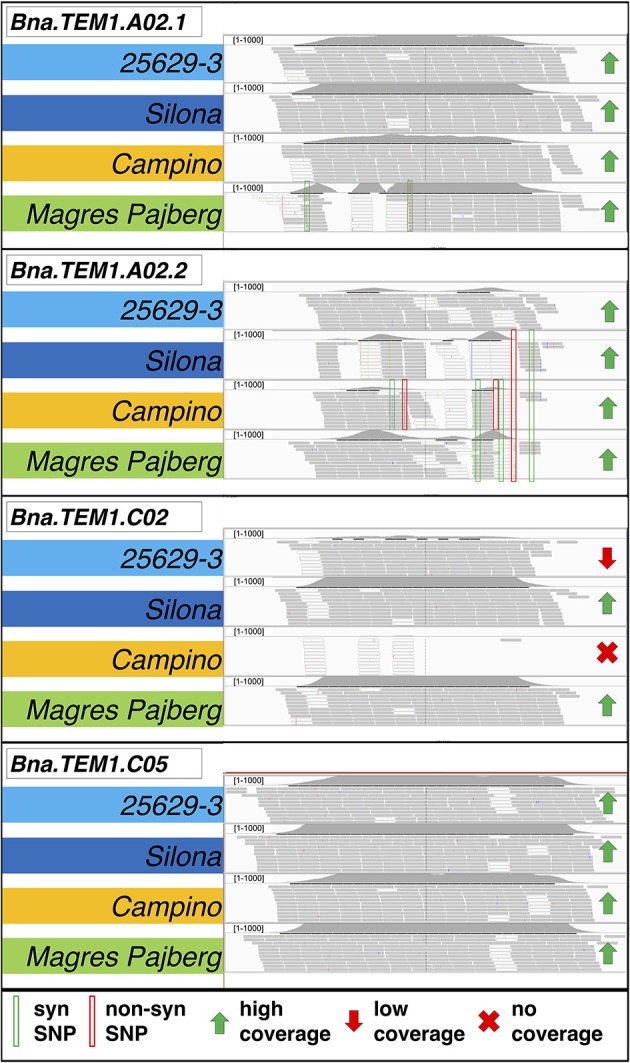
**Mapping of captured sequences from four different *B. napus* morphotypes for paralogs of the gene TEMPRANILLO 1 (Bna.TEM1) on chromosomes A02 (two copies, one showing synonymous SNP variation and one with both non-synonymous and synonymous SNPs), C02 (showing copy-number variation and presence-absence variation) and C05 (no polymorphisms)**. Copy-number variation (CNV) is estimated by variation in the normalized average read density over the entire length of the captured gene sequences of all expected paralogs in the four genotypes. Gray bars indicate uniquely mapped reads, white bar indicate ambiguously mapped reads. Details on the morphotypes of the four accessions are provided in the text.

Figure 2 shows an example for read mapping, depth of coverage estimation and polymorphism detection in four *B. napus* homologs of the gene *TEMPRANILLO 1 (Bna.TEM1)* on chromosomes A02 (two copies, one showing synonymous SNP variation and the other with both non-synonymous and synonymous SNPs), C02 (showing copy-number variation and presence-absence variation) and C05 (no polymorphisms). Despite the high sequence homology between homologs, use of the *B. napus* reference genome assembly enabled reads to be accurately mapped to their respective homologous locus, simplifying the detection of locus-specific sequence polymorphisms and allowing estimation of CNV from the average sequence coverage at each expected locus.

### SNP calling

The results of the SNP calling are summarized in Table 2. After alignment with SOAP2, a total of 17,316 SNPs with a minimum read depth of 10 were called in the total dataset. The targeted region harbored 4269 SNPs, referred to here as target SNPs, resulting in average of 1 SNP per 144 nucleotides. Heterozygous hemi-SNPs representing multiple homologous loci made up 14–27% of the target SNPs, indicating mapping difficulties within duplicated or highly homologous gene regions. For subsequent analyses of potential functional mutations in the target sequences, only true homozygous SNPs in individual target gene loci were considered.

**Table 2:** **High-quality SNPs called within the total enriched sequences (total SNPs) and the targeted gene sequences (target SNPs), respectively**.

**Type of SNP**	**25629-3**	**Silona**	**Campino**	**Magres Pajberg**
Total SNPs, homozygous	2772	3974	4730	5849
Total SNPs, heterozygous	5259	4116	4283	4836
Total SNPs, homozygous (%)	16.01	22.95	27.32	33.78
Total SNPs, heterozygous (%)	30.37	23.77	24.73	27.93
Target SNPs, homozygous	546	990	1351	1538
Target SNPs, heterozygous	1145	599	813	771
Target SNPs, homozygous (%)	12.79	23.19	31.65	36.03
Target SNPs, heterozygous (%)	26.82	14.03	19.04	18.06

A SNP was called when one of the four test genotypes carried an alternative nucleotide to the reference genotype Darmor-bzh in all mapped reads covering a given target nucleotide position, with a minimum of 10 reads.

### Detected sequence variation

Two or more copies of all targeted genes were recovered by the sequence capture, matching BLAST positions of all known homologs in the *B. napus* Darmor-bzh reference genome. In total we identified 160 individual homologs/paralogs for the 29 genes of the target panel. Of these, 23 sequences could not be translated *in silico* to proteins matching database records for *A. thaliana*, *B. rapa*, *B. oleracea*, or *B. napus*, and/or could not be uniquely mapped to a *B. oleracea* or *B. rapa* CDS database. Therein, we found 10 copies not translating to protein at all according to our prediction with GENSCAN, therefore they might be non-functional paralogs. Another 12 copies translated to fragmented or meaningless peptide *in silico*, having no (5 copies) or no unique hit to the respective CDS databases (7 copies). One copy was predicted to translate to meaningless peptide, but had a respective hit in the *B. rapa* CDS database. These copies were also considered non-functional. A further four copies had high homology but were partially missing in the reference genome assembly. 120 copies were captured over their full coding length, while 13 copies only translated to parts of the expected protein. **Figure 6** and Figure S1 show the relative positions of all homologs between *A. thaliana*, *B. rapa*, *B. oleracea*, and *B. napus*. Considering the expected copy number based on the *B. rapa* and *B. oleracea* genomes, a total of 9 copies were lost, whereas 28 (including the 23 non-functional copies) were duplicated. This suggests that only 5 of the new gene duplications were functional, but also that relatively few duplicated paralogs of flowering time regulatory genes have been lost in *B. napus* after polyploidization. Considering all functional copies, this gives a ratio of 1.9:1 comparing the tetraploid with the diploid genomes, representing a 3% change to the expected 2:1 ratio (**Figure 6**, Figure S1).

Comparisons with gene expression data from the semi-winter *B. napus* variety “Ningyou 7” for different time points and treatments suggest that all of the loci we captured and considered functional are expressed in *B. napus* [Carlos Molina, Christian Albrechts University, Kiel, Germany, unpublished data]. Because of its homology to *CO*, the oligonucleotide baits also captured four *B. napus* homologs of the gene *CO-like 2*, although this gene was not included in the target panel. The four captured *Bna.CO-like 2* homologs were therefore included in the further analysis of variation.

DNA sequence variation was detected in 104 of the captured gene sequences. As expected, SNP variation was most predominant, with high-confidence SNPs being observed in 102 of the 104 variable genes. High-confidence CNV was observed at 7 gene loci, with one locus showing PAV. InDels were not detected by SOAPindel within our target regions, therefore no frameshifts are expected in this dataset. A comparative mapping with the software Bowtie (using default settings) showed InDels only in regions of very low mapping quality, so we considered them to be mapping errors.

**Figure 3:**
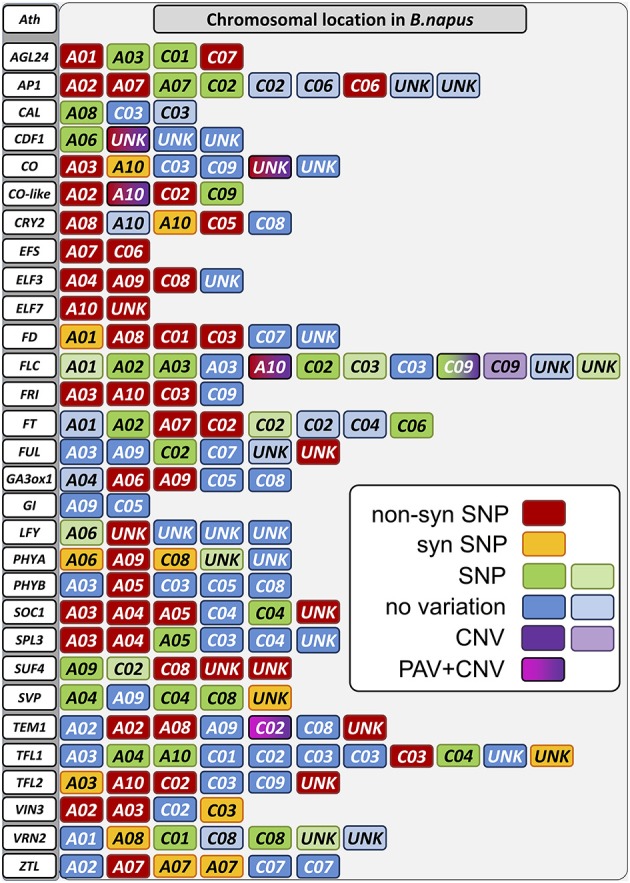
**Sequence variation in detected copies of the panel of target genes**. Chromosomes on which copies of the target genes were detected are colored. Sequence variation is indicated by color according to the legend. A combination of different types of variation at a single locus is shown by a color change within a box. Lighter colors as shown in the legend indicate that this copy is expected to be non-functional.

**Figure 4:**
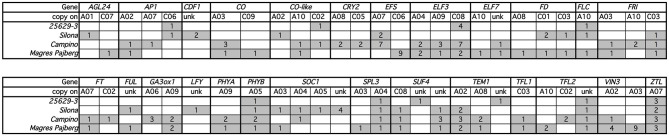
**Distribution of non-synonymous SNPs (gray) in copies of target genes from four diverse *Brassica napus* accessions with different morphophysiological flowering attributes (see text for details)**. Unk, unknown chromosome position.

A total of 313 SNPs were located in exons of the captured genes. Out of these, 188 were synonymous, whereas 125 changed the amino acid sequence in one or more genotypes (Figures 3, 4). An amino acid change in at least one of the genotypes was predicted by 54 of the 141 functional target gene copies. The winter oilseed rape genotype 25629-3, belonging to the same eco-geographical flowering morphotype as the winter rapeseed Darmor-Bzh, differed from the Darmor-Bzh reference genome in only 10 gene copies with non-synonymous mutations. In contrast, the early-flowering fodder rapeseed Silona showed 21 gene loci with non-synonymous SNPs, while 31 loci with non-synonymous SNPs were detected in the cold-sensitive, day-length dependent spring rapeseed Campino and 35 loci in the swede Magres Pajberg, both of which have low winter-hardiness and flower under longer-day conditions.

A total of 54 paralogs of the target genes showed one or more non-synonymous mutations in the coding sequences of the four sequenced genotypes compared to the winter oilseed rape reference genome sequence. Only four genes (*Bna.CAL, Bna.GI, Bna.SVP, and Bna.VRN2*) showed no nucleotide polymorphisms affecting the amino acid composition of the gene product of any paralog. In all other genes, including gene copies assigned to vernalization (*Bna.EFS, Bna.ELF7, Bna.FLC, Bna.FRI, Bna.SUF4, Bna.TFL2, Bna.VIN3*), photoperiod (*Bna.CO, Bna.CO-like, Bna.CRY2 and Bna.PHYA*), gibberellin (*Bna.GA3ox1*), clock (*Bna.CDF1, Bna.ELF3, Bna.ZTL*) and signaling (*Bna.AGL24, Bna.AP1, Bna.FD, Bna.FT, Bna.FUL, Bna.LFY, Bna.SPL3, Bna.SOC1, Bna.TEM1*, and *Bna.TFL1*), we found potentially functional amino acid modifications in the gene products of at least one homolog/paralog within the four different *B. napus* morphotypes (Figure 4).

As expected, the degree of potentially functional sequence diversity in comparison to the *B. napus* reference genome sequence varied among the four sequenced genotypes in correspondence to their ecophysiolologcal diversification from the winter oilseed rape reference genotype Darmor-Bzh. The winter oilseed rape 25629-3 and the winter-hardy fodder rape Silona showed the lowest degree of non-synonymous SNPs in comparison to Darmor-Bzh, while the spring-type canola genotype Campino and the swede Magres Pajberg showed considerable diversity in comparison to Darmor-Bzh. Campino, which flowers under long-day conditions, showed particularly high rates of non-synonymous mutations in photoperiod module genes, whereas Magres Pajberg was the most divergent from Darmor-Bzh in relation to vernalization, clock and signaling genes.

### CNV and PAV

**Figure 5:**
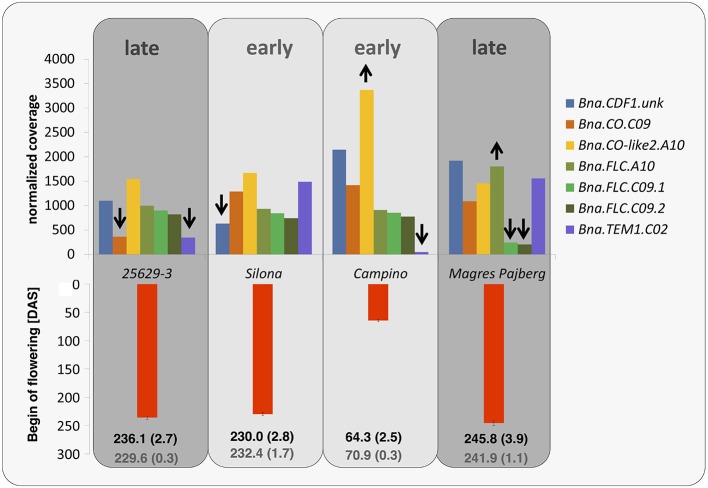
**Normalized coverage for gene copies with copy-number variation or presence-absence variation**. A reduction in copy number compared to the Darmor-Bzh reference genome is indicated by the downwards arrows, an increased copy number by the upwards arrows. The red bars indicate flowering time in days after sowing (DAS), averaged over 3 years and 3 locations in Germany (with standard errors). In the field trials in Germany 25629-3, Silona and Magres Pajberg were tested in autumn-sown trials, whereas the winter-sensitive Campino was grown in a spring-sown trial. The black numbers indicate the genotype mean (with standard error), while the gray numbers indicate the population mean (with standard error).

**Figure 6:**
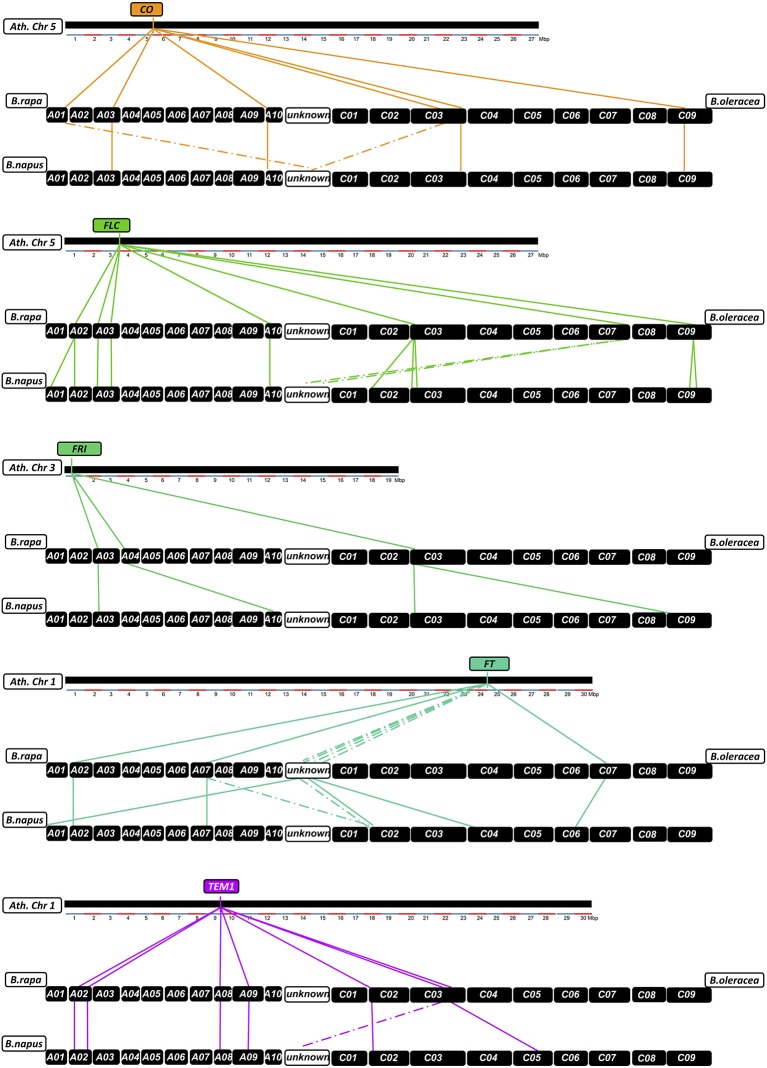
**Relationships between homologs from *Arabidopsis thaliana*, *Brassica rapa*, *Brassica oleracea*, and *Brassica napus* for the flowering time regulatory genes *CO*, *FLC, FRI, FT*, and *TEM1***. Chromosomes are shown as black boxes. Colored lines connect relative chromosomal positions between *A. thaliana* and *B. rapa/B. oleracea* and between *B. rapa/B. oleracea* and *B. napus*. Dotted lines indicate positions that could not be verified by BLAST.

**Figure 7:**
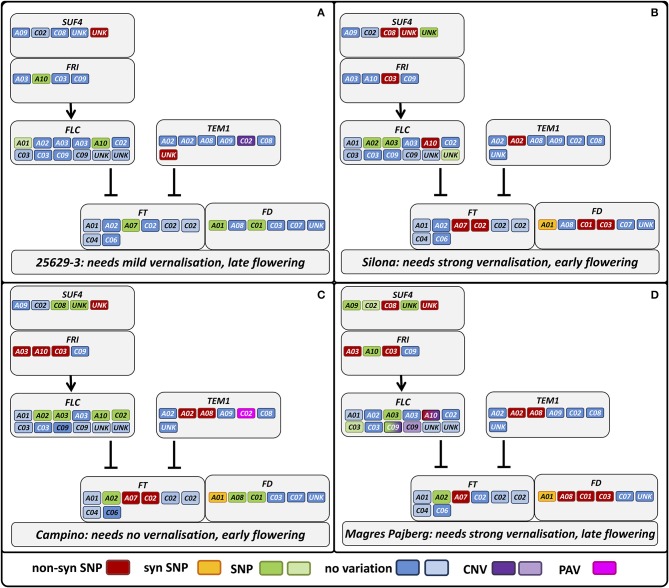
**Differential sequence variation in homologs of genes involved in vernalization response among phenotypically divergent *B. napus* morphotypes**. Gray boxes indicate gene interactions in *A. thaliana*, whereas the colored boxes indicate the respective gene copies in *B. napus* with their respective sequence variation indicated in the legend. Lighter colors in the legend indicate that a copy is predicted to be non-functional. Genotypes: **(A)** winter oilseed rape 25629-3, **(B)** winter fodder rape Silona, **(C)** spring canola Campino, **(D)** swede Magres Pajberg.

In the winter rapeseed genotype 25629-3 we observed reductions in copy number for a copy of *Bna.CO* on chromosome C09 and a copy of *Bna.TEM1* on chromosome C02, respectively. One homolog of *Bna.CDF1*, which was unable to be assigned to a chromosome in the Darmor-Bzh reference genome, was reduced in copy number in the winter fodder rape Silona. On the other hand, the spring canola Campino was found to have a copy number increase in *Bna.CO-like* on chromosome A10, whereas no reads were captured corresponding to *Bna.TEM1* from chromosome C02; we therefore assume that this gene is deleted in Campino. The target coverage for a duplicated *Bna.FLC* locus on chromosome C09 indicated that this locus has been replaced in the swede Magres Pajberg by its homolog from a highly homoeologous chromosome segment on chromosome A10. Homoeologous non-reciprocal translocations are common in the allopolyploid *B. napus* genome (Samans, 2014). Figure 5 shows normalized coverage for the affected copies in each genotype, together with their flowering time. To avoid counting of homoeologous loci (Figure 6, Figure S1) in the CNV estimation, only gene loci for which no heterozygous SNPs were detected were included in the analysis. Figure 7 shows which of these copies carry the respective variation type. *Bna.GI* did not show variation in any of its copies, whereas other genes, (e.g. *Bna.FLC*), exhibited considerable sequence variation at most of their loci.

### FT promoter region

**Figure 8:**
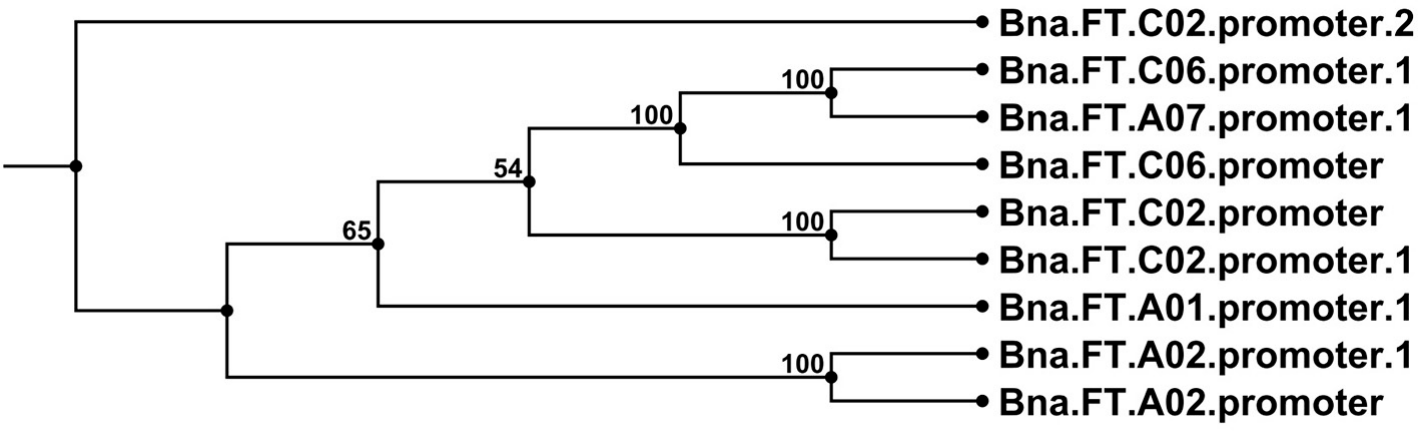
**Neighbor joining tree diagram of promoter sequences from all detected copies of the flowering gene *FT* in comparison with promoters from previously known *Bna.FT* loci retrieved from NCBI**. Sequences detected in the present study are labeled with the suffix 1 or 2, where one or two copies, respectively, were detected on the corresponding chromosome. Setting details can be found in the Materials and Methods Section.

The bait library contained a full-length genomic sequence for *FT* on chromosome A02, including the promoter region. This successfully enriched for the targeted copy including promoter, but also for the promoter regions of FT copies on A01, A07, C02, and C06. Sequence alignments with previously known *B napus FT* promoter sequences (Accession numbers JX193765.1, JX193766.1, and JX193767.1) revealed that the promoter of the newly detected *FT* locus on chromosome A07 is closely related to that of the locus on chromosome C06, whereas the other two newly detected *FT* promoters on chromosomes A01 and C02 diverge from all previously known *FT* promoter sequences (Figure 8). Interestingly, all detected *FT* promoter regions contained considerable SNP variation, with a total of 7 SNPs detected in the promoter region of *Bna.FT.A01*, 9 in *Bna.FT.*A02, 4 in *Bna.FT.*A07, 14 each in *Bna.FT.*C02_1 and *Bna.FT.*C02_2 and 21 in *Bna.FT.*C06. The promoter regions for two further FT copies on C02 and C04 were not detected. Since both are considered non-functional, this strengthens the hypothesis that both of these paralogs are pseudogenes.

## Discussion

Polyploidization was a major driver of crop evolution and many important crop plants are polyploids (e.g., wheat, cotton, sugarcane, potato, rapeseed). This is a major restriction for knowledge transfer from well-studied model plants to crops. The high number of gene copies complicates model development for important traits, in particular for regulation of complex traits like timing of reproduction. The first step of elucidating the interplay between different gene copies of a regulation module is an assessment of their number and sequence variation. The recent allopolyploid crop *B. napus* is an excellent model to study the influence of gene copy number and sequence variation on trait expression for two reasons: (1) the interspecific hybridization arose only a few thousand years ago and the ancestor genomes are still basically intact (Parkin et al., 1995), and (2) the close relationship to the model plant *A. thaliana* and the recently sequenced *B. rapa* allows for comparisons between gene models and crop sequences. Gene cloning strategies and array technologies depend on very specific sequence information, whereas whole-genome sequencing cannot always provide the appropriate coverage for assessment of copy-number variation. Therefore we chose an in-solution hybridization sequence capture approach, aiming to detect all present copies of the targeted flowering time regulatory genes and mine for their variation in number and sequence.

We developed a RNA oligonucleotide pool designed to capture a core set of 29 flowering time genes in *B. napus*. This enabled us to enrich all 164 copies expected from the draft *B. napus* reference genome, proving the value of this technique to capture sequence variants across complex regulatory modules like the flowering time gene network.

All in all, 124 copies of the target genes were deep-sequenced over their full coding length; a further 17 copies were partially captured. This suggests an average copy number of 4.7, which is close to the average number of 4.4 copies per gene expected over the entire *B. napus* genome (Parkin et al., 2010). Independent expression studies suggested that all of these captured flowering time gene copies are functional and expressed in *B. napus*. This represents a huge expansion of active flowering time regulatory genes in the allopolyploid *B. napus* genome in comparison to *A. thaliana*, where most of these genes are represented by only a single active copy. This expansion creates enormous potential for functional differentiation and regulatory plasticity across all pathways influenced the flowering time gene expression network. The selective potential inferred by this polyploidization-induced expansion in flowering-time genes can be speculated to have had a major impact on the natural and artificial selection of different ecophysiological morphotypes of *B. napus*, leading to their differential adaptation into the various cultivated forms.

Compared to whole genome sequencing data, alignment rates from our sequence capture data were high; only a low percentage of around 13% of the reads aligned non-uniquely. Enrichment was successful with an enrichment factor of more than 760x, indicating that baits developed from closely related species are able to efficiently enrich selected regions in *B. napus*; similar efficiency of sequence capture across close relatives was also shown in other species (Vallender, 2011; Bundock et al., 2012; Jupe et al., 2013; Mascher et al., 2013). Sensitivity and specificity were comparably low, with values of 78–81% and 50 to 52%, respectively (Mamanova et al., 2010). Lower values for sensitivity in case of multiplexed samples are reported (Mascher et al., 2013), so the lowered sensitivity may be attributed to multiplexing. Specificity in this case suffers from the artificial definition of the target (see Methods).

Comparing the detected copies to their ancestor genomes, we found only a 3% change in the expected ratio (considering only functional copies) of *B. napus* gene copies compared to the average copy number in the progenitor genomes. This is only a minor change in gene number compared to other polyploids. In wheat, the ratio of the hexaploid gene content compared to one of the diploid progenitors was 2.5–2.7:1, representing a 10–16% change (Brenchley et al., 2012). This illustrates that *B.napus* speciation is only a very recent event (Allender and King, 2010). The retention of functional copies provides more possibilities to introduce variation. More gene variants allow successful selection and adaptation in a wider range of environments, as non-functional copies can be replaced by functional homologs without loss of function. Moreover, environmental-specific beneficial alleles can exist at the same time, allowing for accumulation of a high adaptation potential. In *B. napus* the flexibility achieved by copy number expansion of flowering time regulatory genes is the basis for the great variation among different morphotypes in flowering time as well as in cold adaptation, winter hardiness and vernalization requirement.

We also report 14% non-functional copies, whereas some genes are more affected by non-functional copies than others. For example, we found 5 copies of *FLC* either fragmented or not expressed or both. *FLC* pseudogenes were reported for *Brassica oleracea* (*BoFLC4* and *BoFLC5)* (Razi et al., 2008) and a number of seven functional *BnFLC* copies was already estimated by others (Schranz et al., 2002; Pires et al., 2004).

Another important base for adaptation is copy number variation (CNV) (Żmieńko et al., 2013). We observed CNV for the genes *Bna.CDF1.unk, Bna.CO.C09, Bna.CO-like2.A10, Bna.FLC.A10, Bna.FLC.C09*, and *Bna.TEM1.C02*, and presence-absence variation for *Bna.TEM1.C02*. This variation may have a strong influence on determination of flowering time in the respective morphotypes. For example, the winter oilseed rape 25629-3 is late flowering, winter hardy and needs mild vernalization. We would therefore expect a lower copy number of floral enhancers or a higher copy number of flowering repressors. Indeed, 25629-3 showed a copy-number reduction for *Bna.CO.C09*. The coverage differences in *Bna.TEM1.C02* further suggest that *2*5629-3 only possesses one copy of this locus, in contrast to the early flowering fodder rape Silona and the winter-sensitive Magres Pajberg.

*CO* is a central day length regulator necessary for flowering transition. Accumulation of the CO protein is crucial for flowering initiation (reviewed in Jaeger, 2008; Jung and Muller, 2009; Andrés and Coupland, 2012). In the case of 25629-3, the lower number of *Bna.CO* copies may relate to its late flowering behavior, as a lower number of transcripts can be synthesized at the same time.

*TEM1* is known to bind to the 5′UTR region of *FT*, therefore repressing flowering (Castillejo and Pelaz, 2008). *TEM1* itself seems to be regulated by *FLC* (Deng et al., 2011) and *AP1* (Kaufmann et al., 2010). It has been assumed that the ratio of *CO/TEM* is decisive for *FT* expression in *A. thaliana* (Castillejo and Pelaz, 2008). In the present study, *Bna.TEM1.C02* was found to be absent in Campino, whereas two copies were observed in Silona and Magres Pajberg compared to the single copy found in Darmor-bzh and 25692-3, respectively (see also Figure 7). Considering that all paralogous loci are presumed to be expressed, this corresponds to a *Bna.CO/Bna.TEM1* ratio of 5:6 in 25629-3, 6:7 in Silona and Magres Pajberg, and 6:6 in Campino. Assuming gene dosage effects, these differences might be expected to accordingly influence the vernalization requirement via differential repression of *FT* expression. Correspondingly, Campino is a spring type without need for vernalization, making photoperiod signaling more important than pre-winter repression of flowering. 25692-3 and Darmor-bzh are both winter types with mild vernalization requirement, presumably facilitated by the single copies of *Bna.CO.C09* and *Bna.TEM1.C02*, respectively. On the other hand, *Bna.TEM1.C02* seems to be duplicated in lines with stronger vernalization requirement (Figure 7). Therefore, the cold signal needs to be stronger to overcome the stronger repression.

In 25629-3,we further detected non-synonymous mutations in copies of *Bna.AP1, Bna.CO-like 2, Bna.ELF3, Bna.FLC, Bna.PHYB, Bna.SPL3, Bna.SUF4, Bna.TEM1, Bna.TFL2*, and *Bna.ZTL.* At present it is still unknown whether these mutations are beneficial or disadvantageous, however it is interesting to note that the non-synonymous mutations mainly affect genes involved in temperature perception (*Bna.ELF3, Bna.SPL3*) and vernalization modulation (*Bna.FLC, Bna.SUF4, Bna.TEM1, Bna.TFL2*). This could reflect differences in winter/spring perception among the different eco-physiological morphotypes; if so, the broad range of affected genes provides considerable potential for natural selection of adaptation traits to different environments, a potential advantage of paralog diversification following allopolyploidization.

Silona is a winter fodder rape with strong vernalization requirement. It flowers slightly later compared to winter oilseed types, but relatively early compared to other winter fodder or kale morphotypes. We found a copy number reduction in a *Bna.CDF1* paralog, on an unmapped scaffold (referred to here as *Bna.CDF1.unk*), which may relate to the early-flowering behavior of Silona. It has been shown that *CDF1* directly downregulates *CO* mRNA levels in Arabidopsis (Srikanth and Schmid, 2011), acting as link between the circadian clock and the photoperiod pathway (Niwa et al., 2007), and therefore can be regarded as flowering repressor. Reduction in *Bna.CDF1* transcript abundance due to a copy-number reduction could thus be expected to reduce floral repression and hence accelerate post-vernalization induction of flowering.

Silona was also found to carry non-synonymous mutations in one copy each of *Bna.AGL24, Bna.AP1, Bna.CDF1, Bna.CO-like 2, Bna.EFS, Bna.FLC, Bna.FRI, Bna.FUL, Bna.LFY, Bna.PHYB, Bna.SPL3, Bna.TEM1, BnaTFL2, BnZTL*, in two copies of *Bna.FD, Bna.SUF4* and in four copies of *Bna.SOC1*. Mutations in *Bna.TEM1, Bna.EFS, Bna.FLC, Bna.FRI* and *Bna.TFL2* may relate to its stronger vernalization requirement than the winter oilseed Darmor-Bzh (as discussed before for the reduced *Bna.TEM1.C02* copy number; Figure 7). On the other hand, there also appears to be a stronger variation in downstream effectors, particularly in *Bna.SOC1. SOC1* is a signal integrator for the vernalization, photoperiod and GA signaling pathways and a direct regulator of LFY (Lee and Lee, 2010). As such *SOC1* can therefore be regarded as a floral activator. The results seen here support the assumption that the flowering time shift between earlier-flowering winter oilseed forms and later flowering, leafier winter fodder rape is more likely to be due to mutations in the effector pathways, with only slight modifications to be expected in the input pathways.

Campino is a vernalization-independent, early-flowering spring oilseed rape. We therefore would expect large differences in vernalization genes. As the vegetation period is shifted by 6–8 weeks in spring types compared to winter types, it is necessary for the plants to adapt to warmer and longer days, so we also expect differences in photoperiod and temperature signaling pathway genes. The change from winter to spring behavior in *A. thaliana* is known to be caused by a mutation in either *FRI* or *FLC* or both (Choi et al., 2011). *FRI* is the main activator for *FLC*, while *FLC* is the major flowering repressor before vernalization (Choi et al., 2011). *Bna.FRI* has already been found to play a central role for variation in morphotype, not only for vernalization (Wang et al., 2011a). Correspondingly, we found 3 *Bna.FRI* paralogs carrying mutations in Campino in comparison to the winter rapeseed Darmor-Bzh, whereas only two *Bna.FRI* paralogs differed in Magres Pajberg and one in Silona (Figure 7). Campino is also the only genotype which does not show a mutation in the *Bna.FLC.A10*. This means that 25692-3, Silona and Magres Pajberg share an allele different from Darmor-Bzh and Campino in *Bn.FLC.A10*. It may be concluded that the Darmor-Bzh/Campino allele is less functional than the other, or, more generally, that the *Bna.FLC.A10* copy is not decisive for flowering time determination. As *Bna.FLC.A10* was found to be associated with vernalization behavior, this might be ruled out (Hou et al., 2012). As discussed before, presence-absence variation of *Bn.TEM1.C02* may also play a role in the change to the annual morphotype. As expected, these differences are also accompanied not only by further mutations in vernalization-related genes (*Bna.VIN3, Bna.TEM1, Bna.EFS Bna.ELF7, Bna.SUF4, Bna.TEM1, Bna.TFL2, Bna.VIN3*), but also by numerous mutated sequences in genes from the photoperiod (*Bna.CO, Bna.CO-like 2, Bna.CRY2, Bna.PHYA, Bna.PHYB, Bna.ZTL*) and temperature signaling pathways (*Bna.SPL3*, three copies of *Bna.ELF3*) along with two copies of *Bna.GA3ox1*.

Magres Pajberg is a swede type belonging to the subspecies *napobrassica*. As such this genotype is typically strongly vernalization-dependent and flowers later than winter-type oilseed forms. Compared to the winter line Darmor-bzh, we detected wide sequence variation in Magres Pajberg affecting all pathways under study. Vernalization genes were particularly affected, along with flowering activators (*Bna.VIN3, Bna.TFL2*) and repressors (*Bna.EFS, Bna.ELF7, Bna.FRI, Bna.FLC, Bna.SUF4, Bna.TEM1*). We found further mutations in gene copies from the photoperiod pathway (*Bna.CO, Bna.CO-like 2, Bna.PHYA, Bna.PHYB*), gibberellin synthesis (*Bna.GA3ox1*), temperature signaling (*Bna.SPL3, Bna.ELF3*), the central signaling molecules (*Bna.FT, Bna.FD*) and downstream effectors (*Bna.AGL24, Bna.AP1, Bna.FUL, Bna.SOC1, Bna.TFL1*). We further observed a copy number reduction affecting two *Bna.FLC* paralogs on chromosome C09, which is mirrored by a corresponding copy number increase on A10. This suggests that one of the copies on C09 may have been replaced by a duplication of a locus originating from A10, a widespread effect of polyploidization in *B. napus* caused by homoeologous recombination (Gaeta et al., 2007). A comparison with genome-wide sequence data from different *B. napus* lines showed that these chromosome regions are indeed subject to homoeologous chromosome exchanges in resynthesized *B. napus* (Samans, unpublished data). This example underscores the potential of homeoologous chromosome exchanges to generate functionally relevant copy-number variation among important adaptation genes, illustrating the genomic plasticity of polyploid plants and the genetic potential they harbor for both drastic and more subtle modifications in flowering time and related adaptive phenotypes.

## Conclusions

Different *B. napus* morphotypes show considerable sequence and copy number variation in paralogs of central flowering-time regulatory genes. We demonstrated that most flowering time gene copies arising from the ancestor genomes were retained after allopolyploidization, and many of the retained paralogs are still expressed. The consequence is a huge expansion in the number of flowering-related genes in *B. napus* compared to the related model plant, *A. thaliana*, and a correspondingly large increase in the complexity of the gene networks controlling flowering. Duplications during the recent polyploidization of *B. napus* also provide considerable scope for mutations leading to non-functional paralogs or also neofunctionalization. We demonstrate that sequence capture is a highly efficient method to analyse sequence variation for flowering time and other important pathways in polyploid crop species. Applying this technology to genetic mapping populations and breeding materials will allow us to link sequence variation in flowering time regulatory genes to phenotypic variation for flowering and other important agronomic traits.

### Conflict of interest statement

The authors declare that the research was conducted in the absence of any commercial or financial relationships that could be construed as a potential conflict of interest.
