# Supplementary figures and images for: Capturing sequence variation among flowering-time regulatory gene homologs in the allopolyploid crop species Brassica napus
Source: Front Plant Sci. 2014 Aug 25;5:404. doi: 10.3389/fpls.2014.00404 (PMC4142343; doi:10.3389/fpls.2014.00404)

B

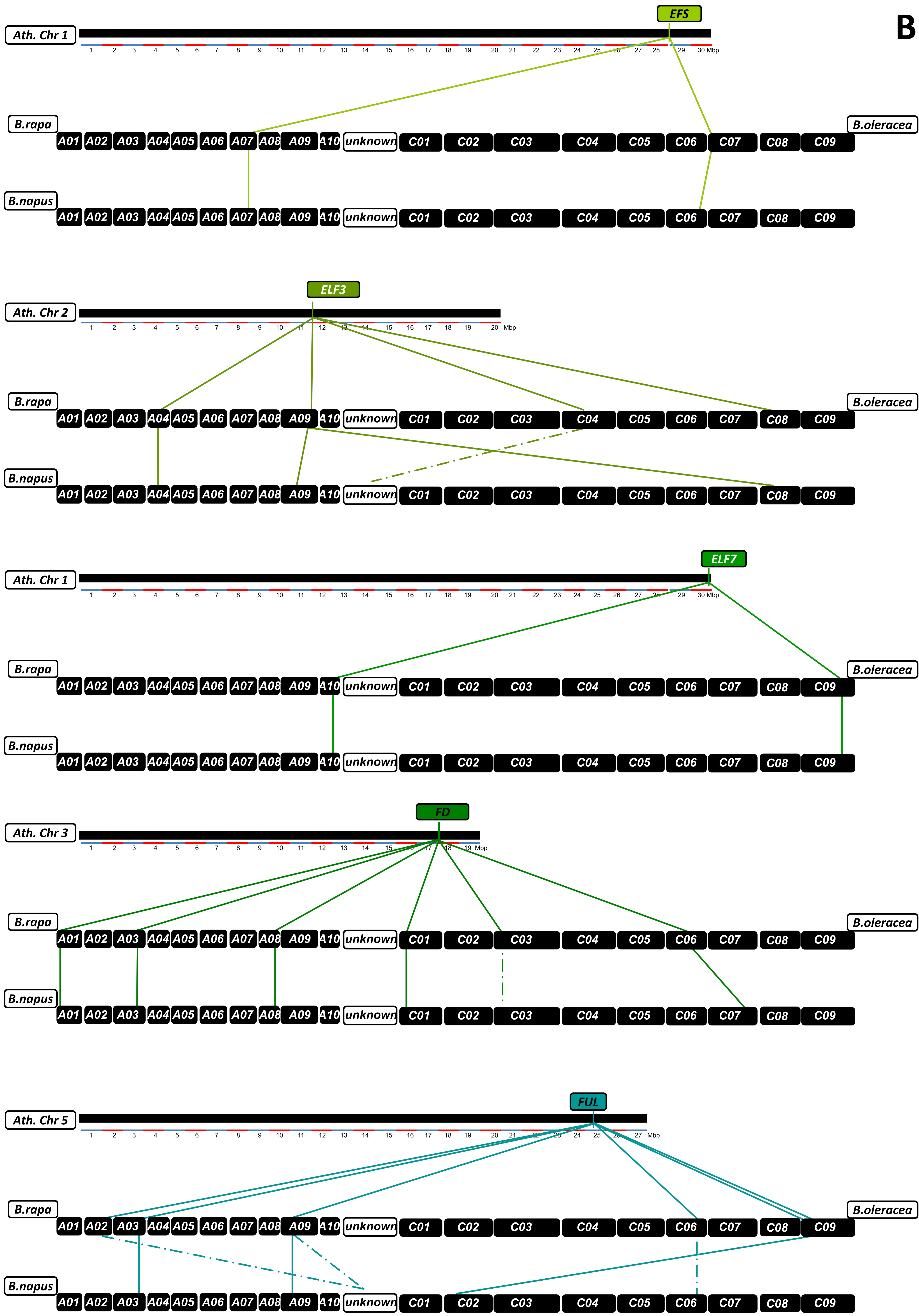

C

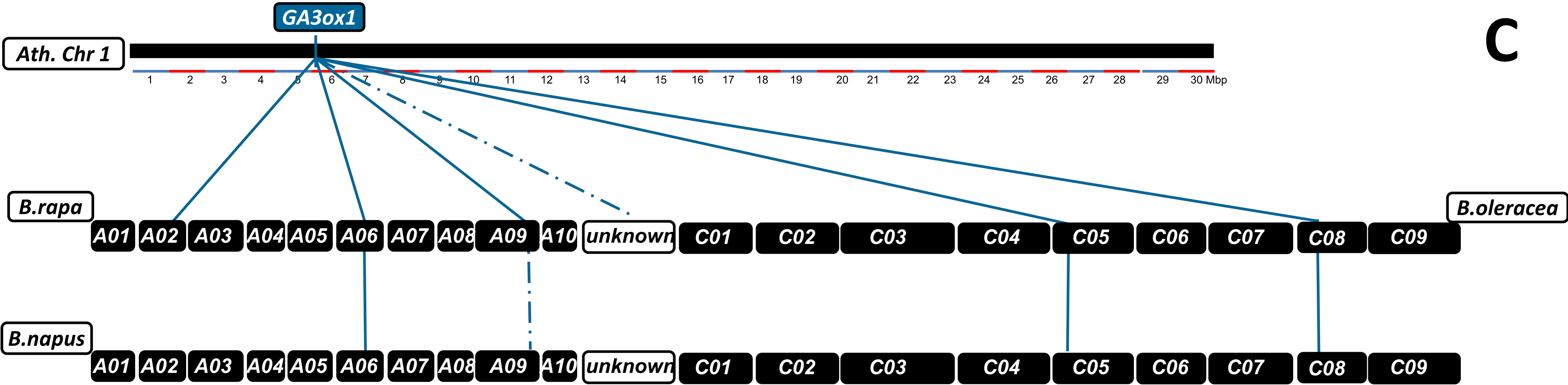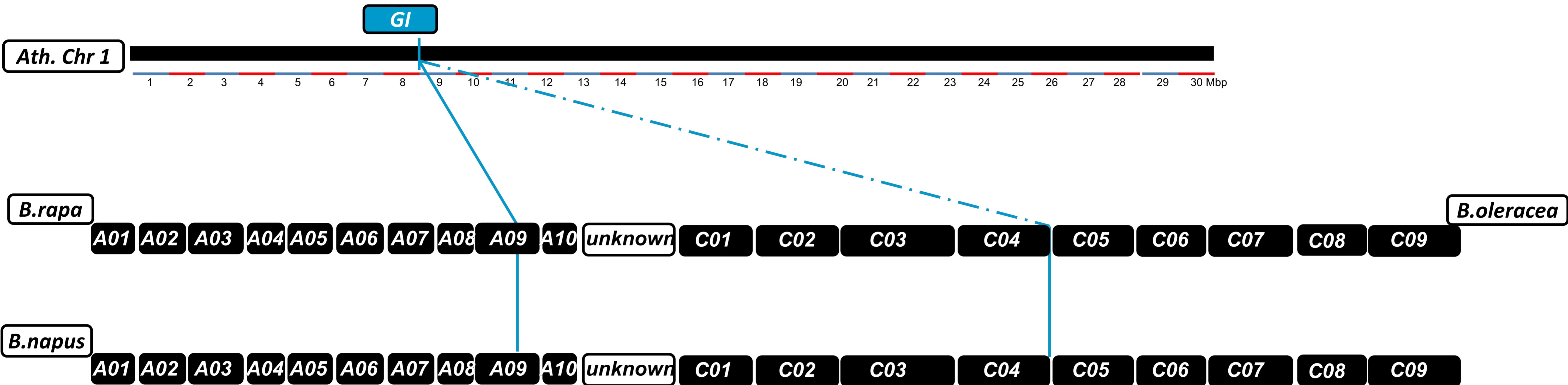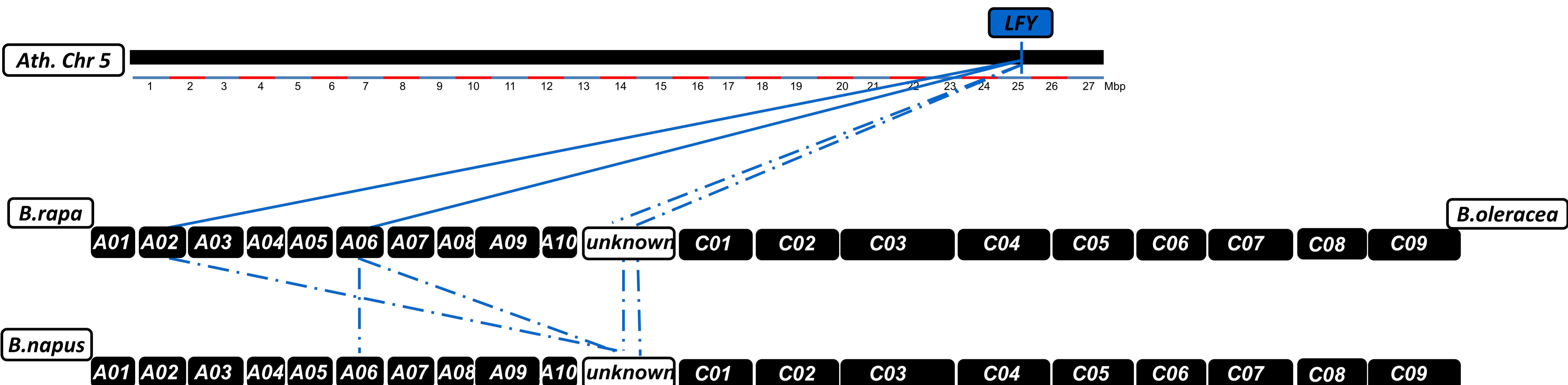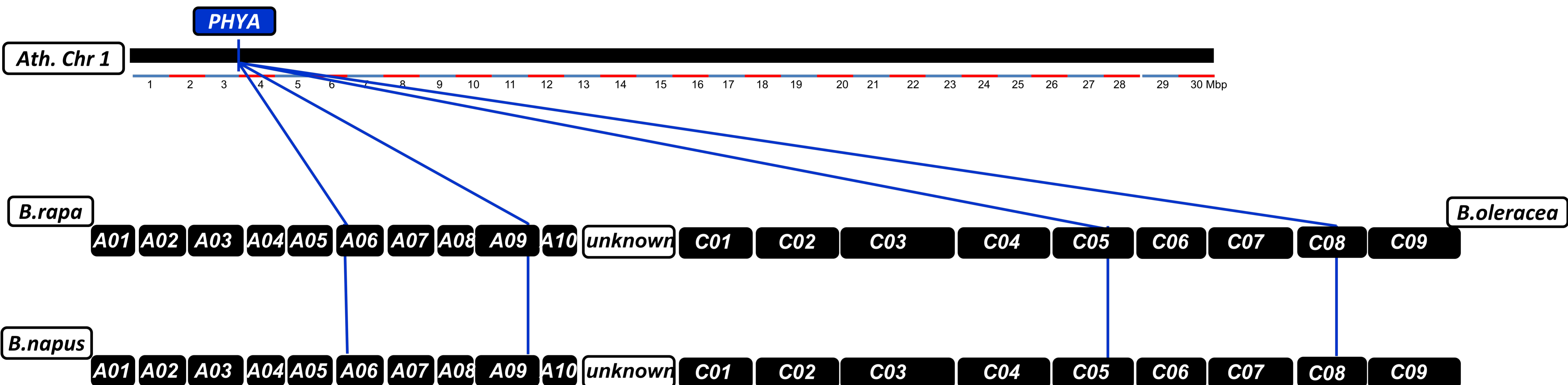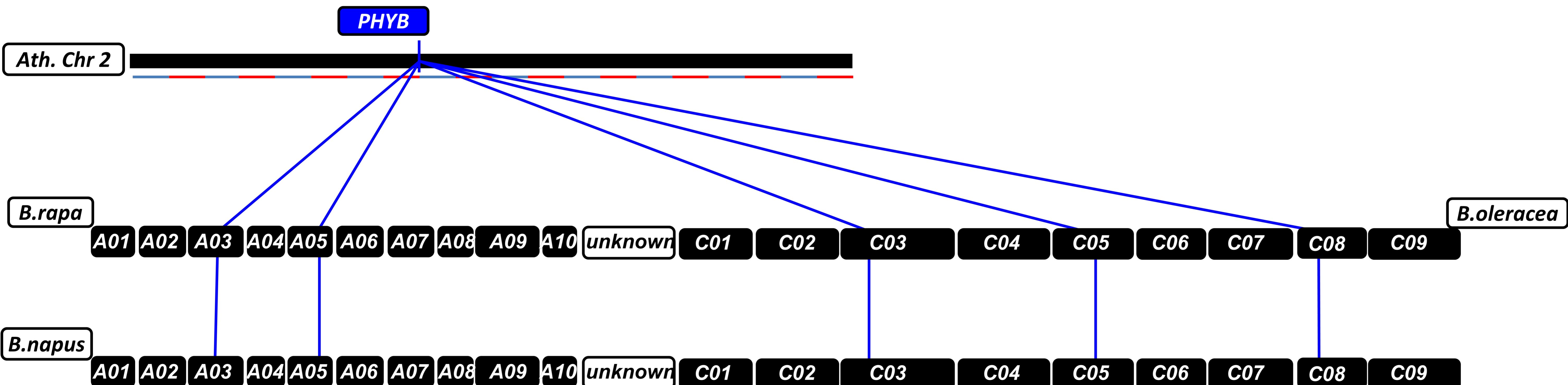

D

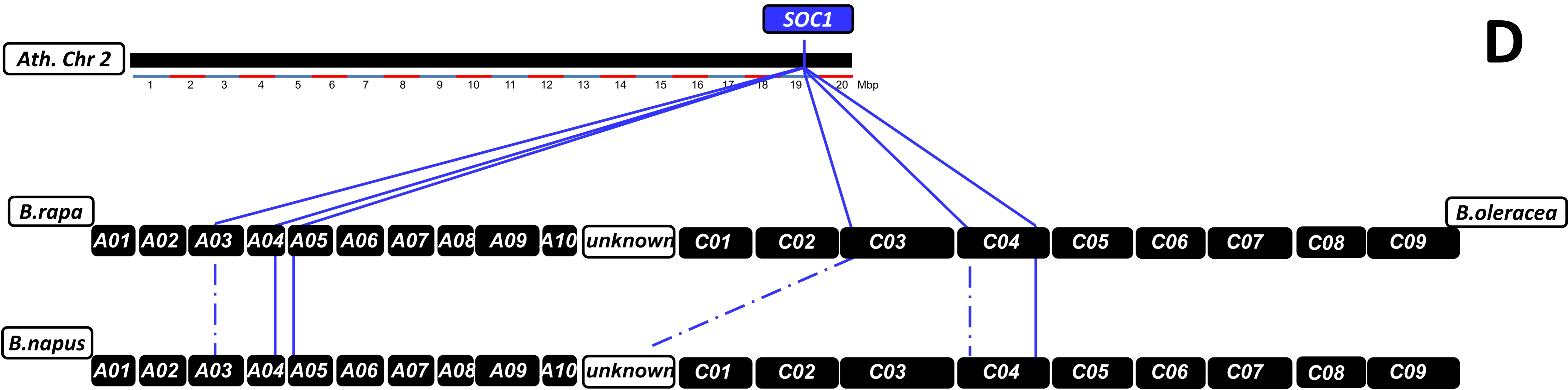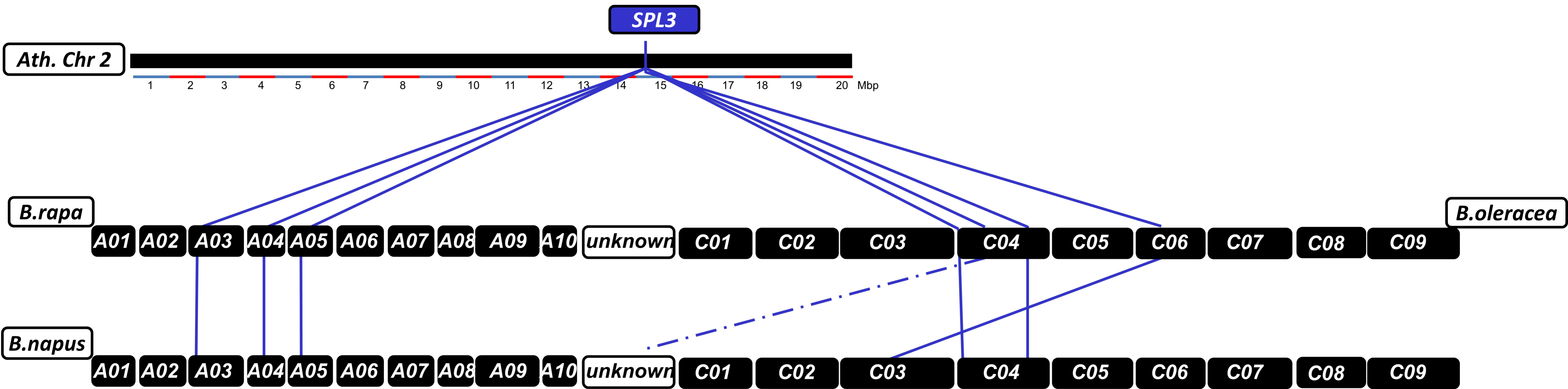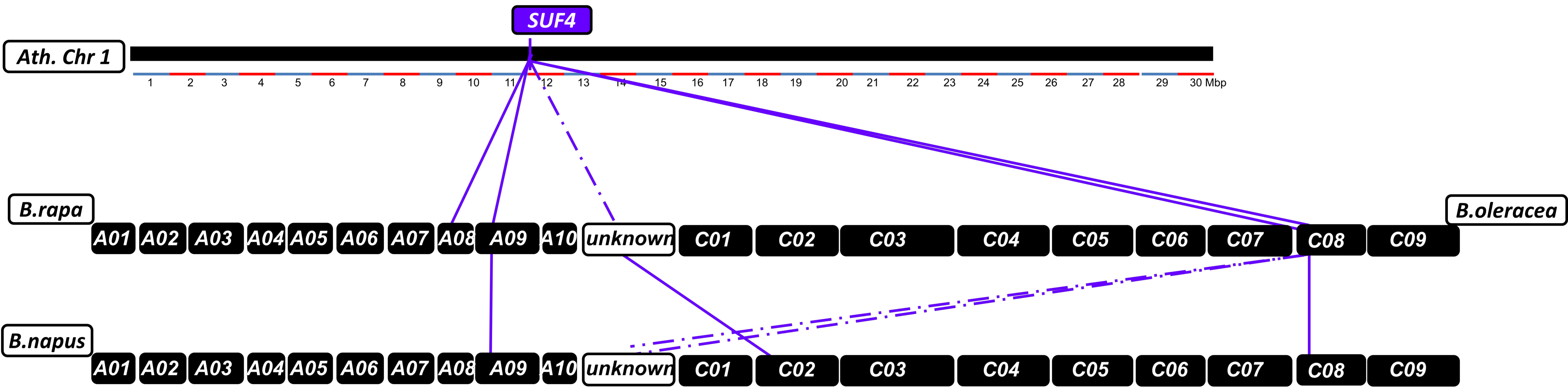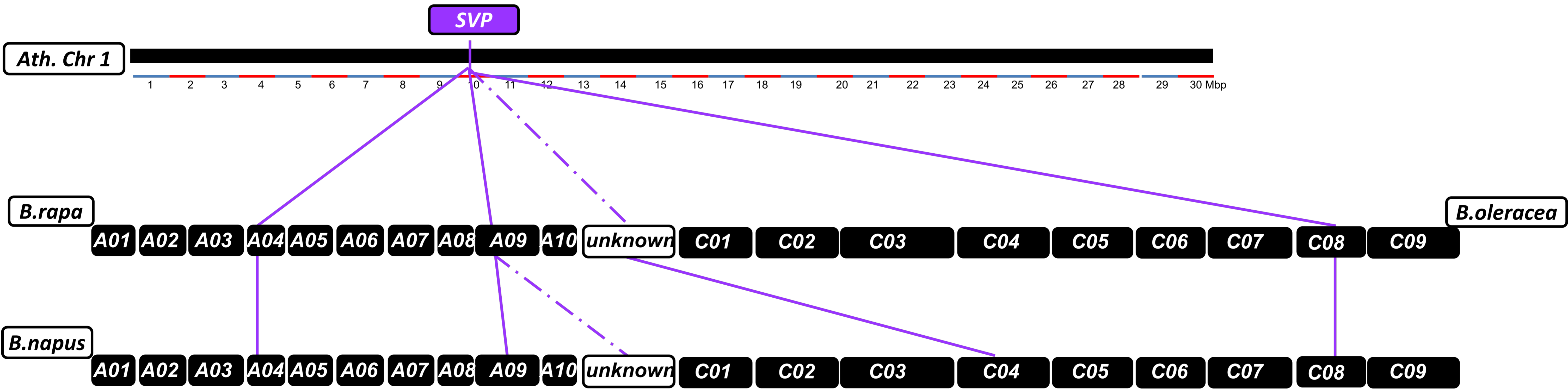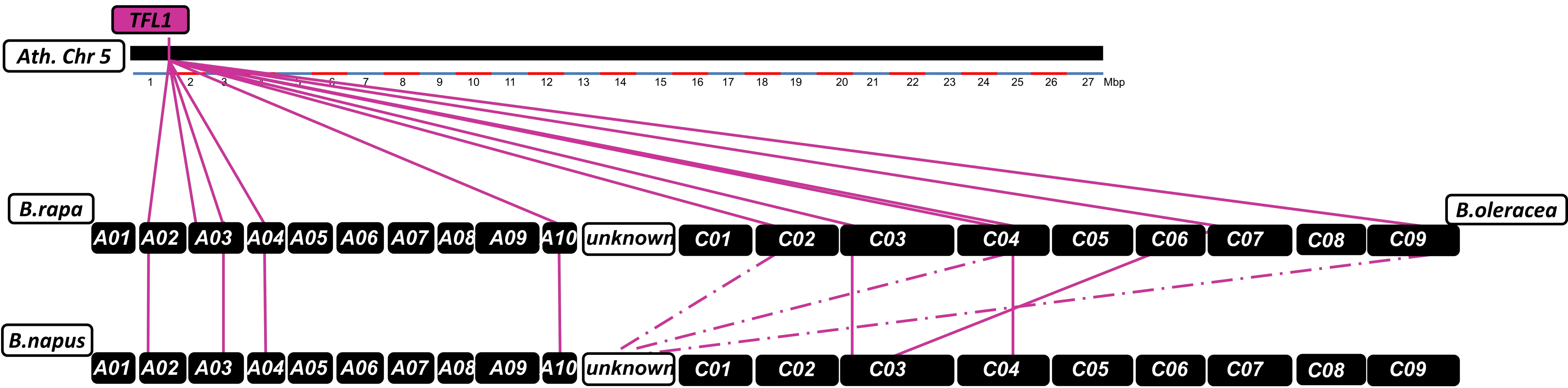

E

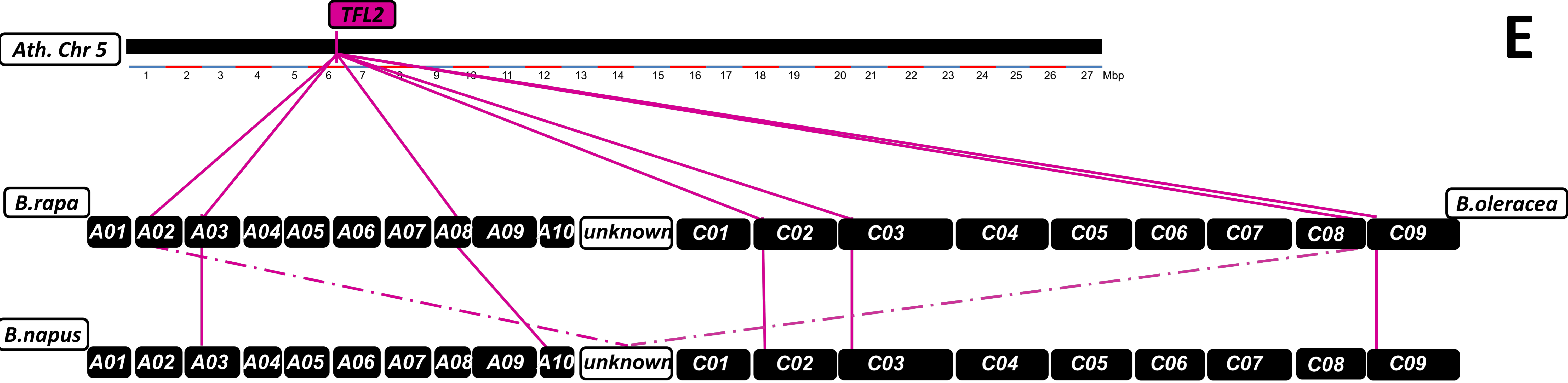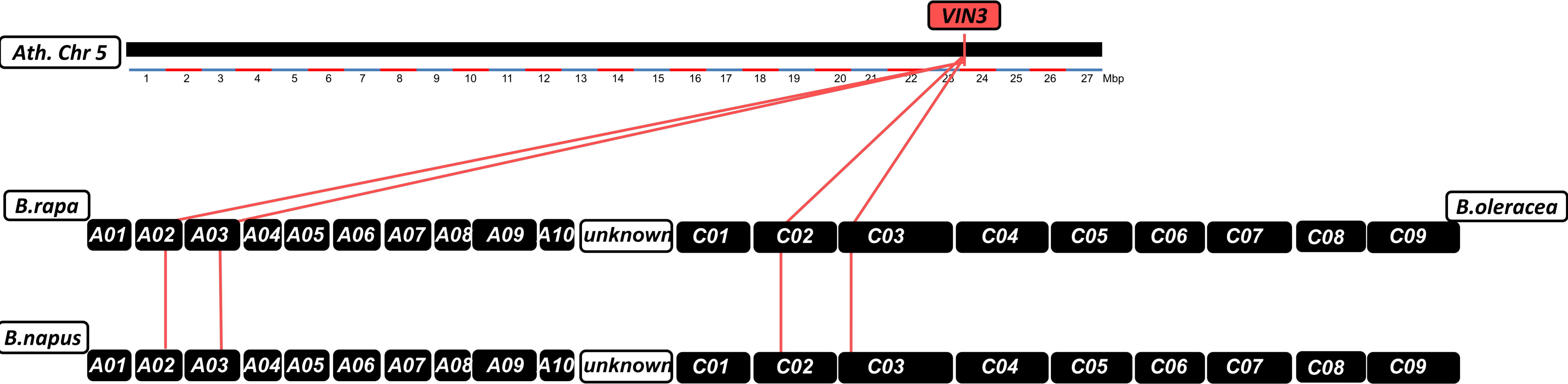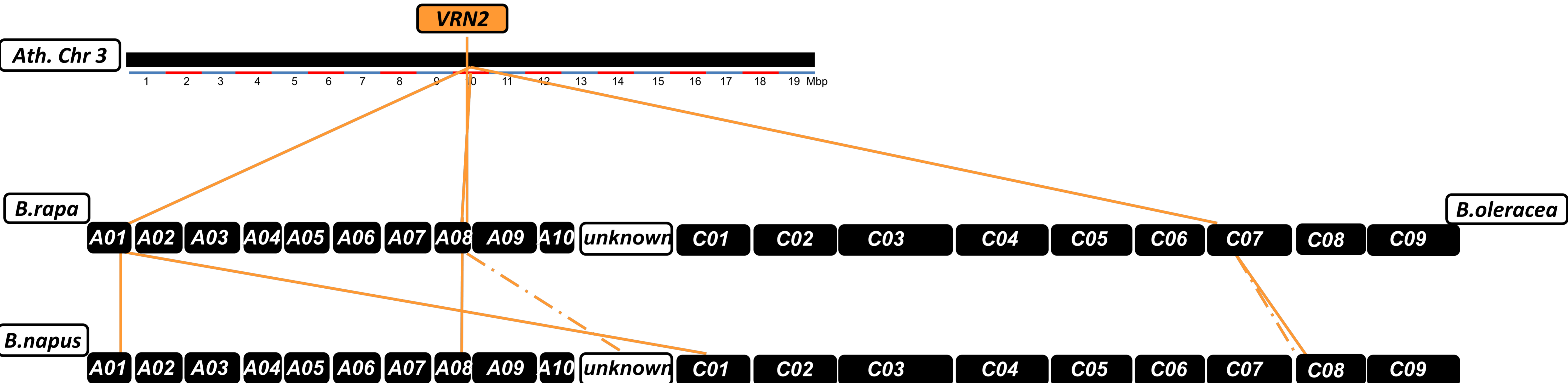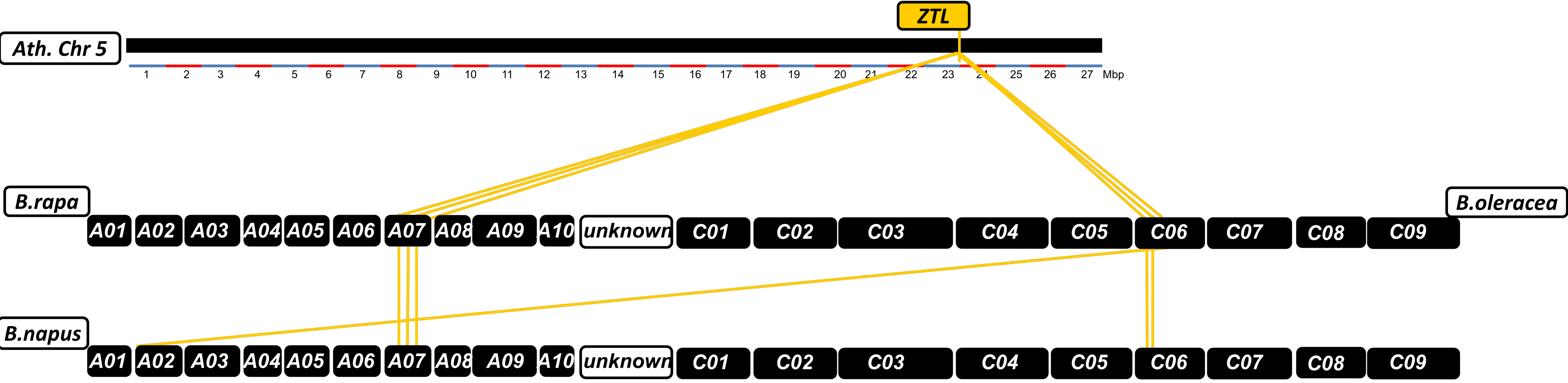

Supplement: Supplementary file 1 [file Presentation1.PDF]
