# Supplementary material for: Capturing sequence variation among flowering-time regulatory gene homologs in the allopolyploid crop species Brassica napus
Source: Front Plant Sci. 2014 Aug 25;5:404. doi: 10.3389/fpls.2014.00404 (PMC4142343; doi:10.3389/fpls.2014.00404)
Supplement: Supplementary file 2 [file DataSheet1.PDF]

Capturing sequence variation among flowering-time regulatory gene homologues in the allopolyploid crop species *Brassica napus*

Sarah Schiessl[1]*, Birgit Samans[1], Bruno Hüttel[2], Richard Reinhard[2], Rod J. Snowdon[1]

[1] Department of Plant Breeding, IFZ Research Centre for Biosystems, Land Use and Nutrition, Justus Liebig University, Giessen, Germany
[2] Max Planck Institute for Breeding Research, Cologne, Germany

Supplementary data

Table S1: Summary of raw sequence alignment results (total and relative numbers of reads) with SOAP2 using default settings. Uniquely aligned reads were determined using option r=0.

**Read fraction**	***25629-3***	***Silona***	***Campino***	***Magres Pajberg***
Uniquely aligned reads	3589959	10204269	2884442	11032045
Uniquely aligned reads (%)	73.76	71.05	70.28	68.6
Non-uniquely aligned reads	680488	1985198	567006	2310830
Non-uniquely aligned reads (%)	13.98	13.82	13.81	14.36
Aligned reads, total	4270447	12189467	3451448	13342875
Aligned reads, total (%)	87.74	84.87	84.09	82.96
Total reads	4867006	14362488	4104487	16082569
